# Developmental Stage-Specific Distribution of Macrophages in Mouse Mammary Gland

**DOI:** 10.3389/fcell.2019.00250

**Published:** 2019-10-24

**Authors:** Teneale A. Stewart, Katherine Hughes, David A. Hume, Felicity M. Davis

**Affiliations:** ^1^Faculty of Medicine, Mater Research Institute-The University of Queensland, Brisbane, QLD, Australia; ^2^Translational Research Institute, Brisbane, QLD, Australia; ^3^Department of Veterinary Medicine, University of Cambridge, Cambridge, United Kingdom

**Keywords:** mammary gland, macrophages, development, embryonic mammary stem cells, adult mammary stem cells, stem cell niche

## Abstract

Mammary gland development begins in the embryo and continues throughout the reproductive life of female mammals. Tissue macrophages (Mϕs), dependent on signals from the Mϕ colony stimulating factor 1 receptor (CSF1R), have been shown to regulate the generation, regression and regeneration of this organ, which is central for mammalian offspring survival. However, the distribution of Mϕs in the pre- and post-natal mammary gland, as it undergoes distinct phases of development and regression, is unknown or has been inferred from immunostaining of thin tissue sections. Here, we used optical tissue clearing and 3-dimensional imaging of mammary tissue obtained from *Csf1r-EGFP* mice. Whilst tissue Mϕs were observed at all developmental phases, their abundance, morphology, localization and association with luminal and basal epithelial cells exhibited stage-specific differences. Furthermore, sexual dimorphism was observed at E14.5, when the male mammary bud is severed from the overlying epidermis. These findings provide new insights into the localization and possible functions of heterogeneous tissue Mϕ populations in mammogenesis.

## Introduction

Mammary gland development is phasic, with distinct developmental periods occurring in the embryo, at puberty and during pregnancy/lactation (Watson and Khaled, 2008; Lloyd-Lewis et al., 2017). The formation of the milk lines occurs at approximately embryonic day (E) 10 in mice and within 36 h resolves into five pairs of disk-shaped thickenings known as mammary placodes (Cowin and Wysolmerski, 2010). At around E12.5, mammary placodes invaginate into the dermal mesenchyme forming the mammary buds, which later elongate and invade the fat pad precursor, creating a rudimentary epithelial tree (Cowin and Wysolmerski, 2010; Paine and Lewis, 2017; Lilja et al., 2018). During embryonic development, multipotent mammary stem cells are replaced by unipotent luminal and basal stem/progenitor cells (Lilja et al., 2018; Wuidart et al., 2018), with epithelial cell identities being resolved by E15.5 (Lilja et al., 2018).

Initial postnatal growth of the mammary epithelium is proportional to body size and it is not until puberty that ductal elongation occurs, fueled by proliferation of adult mammary stem/progenitor cells within terminal end bud (TEB) structures (Davis et al., 2016; Lloyd-Lewis et al., 2017, 2018; Paine and Lewis, 2017). Further epithelial expansion occurs during pregnancy to generate the functional (milk-producing) alveolar epithelium (Watson and Khaled, 2008; Davis et al., 2016). With the cessation of infant suckling, alveolar mammary epithelial cells undergo massive programed cell death (a process known as post-lactational involution), returning the mammary gland to a near pre-pregnant state that is capable of supporting future pregnancies (Sargeant et al., 2014; Lloyd-Lewis et al., 2017).

Mϕs are present in all adult tissues (Hume D. et al., 2019). These cells are first and foremost professional phagocytes, but also regulate tissue development, function and dysfunction (Hume, 2015; Naik et al., 2018; Yang et al., 2018). In the normal postnatal mammary gland, Mϕs regulate ductal morphogenesis during puberty (Gouon-Evans et al., 2000; Van Nguyen and Pollard, 2002; Ingman et al., 2006), alveolar budding during ovarian cycling (Chua et al., 2010), alveologenesis in pregnancy (Pollard and Hennighausen, 1994) and tissue remodeling during post-lactational involution (O’Brien et al., 2010, 2012; Hughes et al., 2012), with many of these processes being impaired in mice deficient in tissue Mϕs. Moreover, Mϕs identified by fluorescence-activated cell sorting (FACS) of disaggregated tissue were detected within the embryonic mammary gland by E16.5 and fetal-derived Mϕs were apparently retained and expanded by self-renewal in adult mammary tissue (Jäppinen et al., 2019).

With accumulating evidence demonstrating the dependence of the mammary epithelium on Mϕs at all developmental stages, it is tempting to speculate that tissue-resident Mϕs institute or influence a putative mammary stem cell niche, as has been shown for hematopoietic stem cells (Winkler et al., 2010), intestinal stem cells (Sehgal et al., 2018) and hair follicle stem cells (Castellana et al., 2014; Naik et al., 2018). Indeed, the activity of mammary “stem” or repopulating cells (defined as a subset of basal cells that are capable of recreating the bi-layered mammary epithelium upon limiting dilution transplantation) is reduced when cells are transplanted into the cleared fat pads of Mϕ-depleted recipient mice (Gyorki et al., 2009). More recently, mammary repopulating cells were shown to express a Notch ligand Delta like 1 (DLL1) and *Dll1*-conditional knockout mice showed reduced mammary repopulating activity and lower levels of F4/80^+^ Mϕs (Chakrabarti et al., 2018). Thus, it has been suggested that DLL1-expressing basal cells activate Notch-expressing Mϕs in a reciprocal stem cell-macrophage niche (Chakrabarti et al., 2018; Kannan and Eaves, 2018). Studies revealing developmental stage-dependent distribution of Mϕs in the mammary gland, including their sites of confluence, would provide further evidence for the existence of a stem cell-macrophage niche in this organ and may help to reveal the specific and stage-dependent localization of mammary stem/progenitor cells within the dynamic, bilayered epithelium under physiological conditions. Here, we utilize a fluorescent reporter model and optical tissue clearing techniques to reveal the presence, prevalence and position of Mϕs in the mammary gland at all phases of development.

## Materials and Methods

### Reagents

Neutral buffered formalin (NBF), Quadrol^®^, triethanolamine and 4′,6-diamidino-2-phenylindole (DAPI) dilactate were purchased from Sigma Aldrich. Normal goat serum was purchased from ThermoFisher. Urea and sucrose were purchased from Chem-Supply. Triton-X-100 was purchased from VWR International. The following primary antibodies were used for immunostaining: chicken anti-GFP (Abcam, ab13970, batch #s GR3190550-3 and -12), rat anti-F4/80 (Novus, NB600-404), rat anti-keratin 8 (DSHB, TROMA-I, batch #s 7/7/16 and 30/3/17), rabbit anti-keratin 5 (BioLegend, 905504, batch # B230397) and rabbit anti-SMA (Abcam, ab5694, batch # GR3183259-26). The following secondary antibodies were used: goat anti-chicken Alexa Fluor-488 (ThermoFisher, A21236), goat anti-rat Cy3 (ThermoFisher, A10522) and goat anti-rabbit Alexa Fluor-647 (ThermoFisher, A21245).

### Animal Models

Animal experimentation was carried out in accordance with the *Australian Code for the Care and Use of Animals for Scientific Purposes* and the *Queensland Animal Care and Protection Act (2001)*, with local animal ethics committee approval. Animals were housed in individually ventilated cages with a 12 h light/dark cycle. Food and water were available *ad libitum*. *Csf1r-EGFP* (MacGreen) (Sasmono et al., 2003) mice were a kind gift from A/Prof Allison Pettit (Mater Research Institute-UQ). Mice were maintained as hemizygotes on a C57BL6/J background. C57BL6/J mice were obtained from the Animal Resources Center (Western Australia).

To obtain mammary tissue during gestation, female mice were mated and tissue harvested 14.5 days-post-coitus (mean no. embryos: 7; range: 6–9). GFP^+^ embryos (E14.5) were also harvested and analyzed after PCR-sexing. To obtain tissue during lactation, female mice were mated, allowed to litter naturally and lactating mammary tissue harvested on day 10 of lactation. For studies during involution, females were allowed to nurse for 10 days and mammary glands harvested 96 h post forced involution. Litter sizes were not standardized (mean litter size: 7; range: 5–10). Mammary glands from pre-pubertal female GFP^+^ mice (postnatal day 10), pubertal (6.5 weeks) and post-pubertal (12 weeks) were also harvested and analyzed. No estrus staging was performed in these studies. In all mice the 2nd, 3rd, 4th, and 5th mammary glands were excised and fixed as described above; 2nd/3rd and 5th mammary glands were preferentially selected for 3D imaging, owing to their smaller size.

### CUBIC-Based Tissue Clearing and IHC

Tissue clearing was performed as previously optimized and described (Davis et al., 2016; Lloyd-Lewis et al., 2016). Briefly, mammary tissue was spread on foam biopsy pads and fixed for 6–9 h in NBF (10%). Embryos were fixed whole. For CUBIC-based clearing, tissue was immersed in Reagent 1A (Susaki et al., 2014; Lloyd-Lewis et al., 2016) at 37°C for 3 days before washing and blocking in goat serum (10%) in PBS with Triton-X-100 (0.5%) overnight at 4°C. Tissue was incubated in primary antibody in blocking buffer for 4 days and secondary antibody in blocking buffer for 2 days at 4°C. DAPI (5 μg/mL) treatment was performed for 2–3 h at room temperature [omitted for second harmonic generation (SHG)] and tissue was immersed in modified Reagent 2 (Lloyd-Lewis et al., 2016) at 37°C for at least 24 h prior to imaging.

### Immunohistochemistry (FFPE Slides)

IHC on FFPE slides was performed as previously described in detail (Stewart et al., 2019). Wholemount immunostaining using anti-GFP antibody was performed prior to processing for paraffin embedding.

### Microscopy

Immunostained tissue sections were imaged using an Olympus BX63 upright epifluorescence microscope using UPlanSAPO 10 × /0.4, 20 × /0.75, 40 × /0.95, 60 × /1.35, and 100 × /1.35 objective lenses. Immunostained optically cleared tissue was imaged using an Olympus FV3000 laser scanning confocal microscope with UPLSAPO 10 × /0.40, UPLSAPO 20 × /0.75, UPLSAPO 30 × /1.05, and UPLFLN 40 × /0.75 objective lenses. 3D de-noising was performed as previously described (Boulanger et al., 2010). For SHG, images were acquired using a Mai Tai DeepSee multiphoton laser on a Zeiss 710 laser scanning inverted microscope. Visualization and image processing was performed in ImageJ (v1.52e, National Institutes of Health) (Linkert et al., 2010; Schindelin et al., 2012).

## Results

### Mϕs Are Present in the Embryonic Bud and Early Postnatal Gland With Sexually Dimorphic Distribution

Mϕs have never been visualized in the embryonic mammary gland. A recent study by Jäppinen et al. revealed the presence of F4/80^+^ cells in digested mammary tissue by E16.5 by flow cytometry (Jäppinen et al., 2019). However, in the absence of *in situ* imaging, it is currently unclear whether these embryonic Mϕs physically associate with the developing mammary epithelium, as has been observed in the postnatal gland.

To assess Mϕ distribution in 3-dimensions in intact mammary tissue, we used a *Csf1r-EGFP* mouse model (Sasmono et al., 2003), combined with methods for optical tissue clearing and deep tissue imaging (Supplementary Figure S1) (Davis et al., 2016; Lloyd-Lewis et al., 2016, 2018). In this model, green fluorescent protein (GFP) expression in tissues is restricted to monocytes and Mϕs in the developing embryo, starting with yolk sac-derived phagocytes, and in all adult tissues (Sasmono et al., 2003; Hume D. A. et al., 2019). Much lower expression in granulocytes and some B lymphocytes is detectable by FACS, but not in tissues. Multi-color fluorescence immunostaining of tissue sections from mouse spleen confirmed that the majority of GFP^+^ cells were also positive for the Mϕ cell surface marker, F4/80 (Supplementary Figure S2). Previous studies using digested mammary tissue from *Csf1r-EGFP* mice analyzed by flow cytometry have shown that >90% of GFP^+^ cells in the mammary gland react with F4/80 (Chua et al., 2010; Hodson et al., 2013).

**FIGURE 1:**
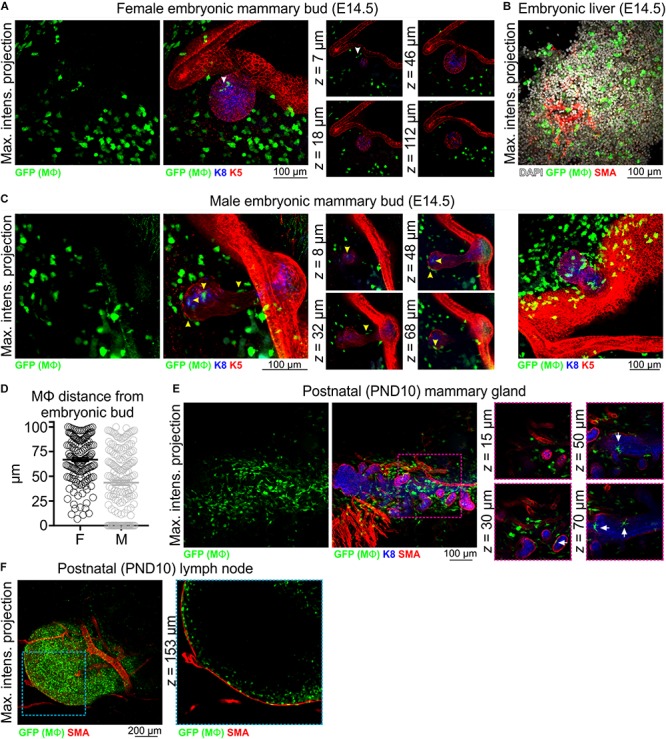
Mϕs in the embryonic and early postnatal mouse mammary gland. Maximum intensity *z*-projection and single optical (*z*) slices of cleared tissue from **(A,B)** embryonic (E14.5) female mice and **(C)** embryonic (E14.5) male mice. **(D)** The distance of Mϕs (within a 100 μm radius) of the female and male embryonic buds. Mϕs contacting the bud or inside of the bud were assigned a value of 0; this was only observed in male embryos. **(E)** Mammary tissue from postnatal day (PND) 10 *Csf1r-EGFP* female mice. **(F)** Inguinal lymph node from PND10 mice showing subcapsular sinus Mϕs. Keratin (K) 8 immunostaining shows K8-positive luminal cells; K5 immunostaining reveals K5-expressing basal cells; smooth muscle actin (SMA) immunostaining reveals basal cells and SMA-positive vessels. White arrowhead in **(A)** points to a Mϕ that appears to be in contact with the embryonic bud in the maximum intensity projection, but is revealed to be positioned in the mammary mesenchyme above the bud in optical slices. Yellow arrowheads in **(C)** point to Mϕs that are in direct contact with the embryonic bud. Arrows in **(E)** point to Mϕs that are in contact with the PND10 mammary epithelium. Images are representative of 3 mice/embryos at each developmental stage.

In 3D image stacks of female *Csf1r-EGFP* embryos, Mϕs were detected in the mammary and dermal mesenchyme surrounding the mammary epithelial bud as early as E14.5 (Figure 1A and Supplementary Figure S3A). As expected (Sasmono et al., 2003), Mϕs were also present in the embryonic liver at this stage (Figure 1B), and it has been suggested that these fetal liver-derived Mϕs contribute extensively to the pool of tissue Mϕs present in the adult gland (Jäppinen et al., 2019). Our data show that Mϕs were positioned adjacent to the embryonic mammary epithelium around the time of lineage segregation (Lilja et al., 2018; Wuidart et al., 2018). Interestingly, although Mϕs were positioned around the embryonic bud, they were rarely observed to directly interact with the developing epithelium of female embryos (Figure 1A and Supplementary Figure S3A). In contrast, Mϕs directly contacted and invaded the mammary bud of male mice at E14.5, the developmental period when the male bud is severed from the overlying epidermis in mice and begins to regress (Figures 1C,D and Supplementary Figure S3B) (Dunbar et al., 1999; Heuberger et al., 2006; Cowin and Wysolmerski, 2010). Mammary Mϕs were also observed in the early postnatal period in female mice (Figures 1E,F). By this stage, however, Mϕs were positioned around and inside of this rudimentary structure, apparently interacting with the epithelium (Figure 1E).

### Mϕs Envelope and Infiltrate the Elongating Terminal End Bud During Ductal Morphogenesis

Mϕs are essential for normal ductal morphogenesis during puberty (Gouon-Evans et al., 2000; Van Nguyen and Pollard, 2002; Ingman et al., 2006). Pre-pubertal leukocyte depletion using sub-lethal γ-irradiation is associated with impaired ductal development and in Mϕ-deficient *Csf1^*op*^/Csf1^*op*^* mice, misshapen TEBs fail to properly invade the mammary fat pad at the rate observed in age-matched controls (Gouon-Evans et al., 2000; Van Nguyen and Pollard, 2002; Ingman et al., 2006). Previous studies analyzing Mϕ density and distribution in mouse mammary tissue sections have shown recruitment of F4/80^+^ Mϕs to the pubertal epithelium and their convergence around the neck of TEBs (Gouon-Evans et al., 2000; Schwertfeger et al., 2006), where adult mammary stem/progenitor cells are thought to reside (Sreekumar et al., 2015; Lloyd-Lewis et al., 2017).

**FIGURE 2:**
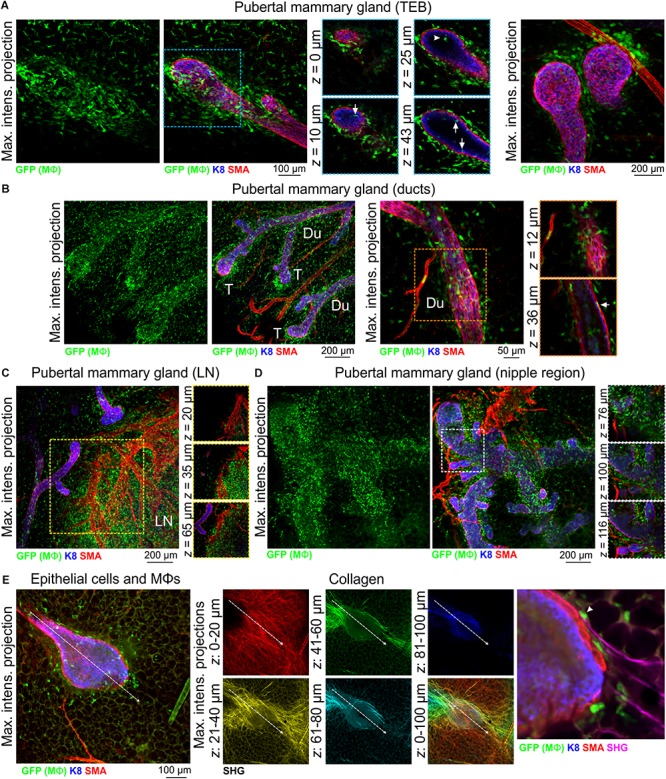
Mϕs in the mammary glands of pubertal virgin mice. Maximum intensity *z*-projection and single optical (*z*) slices of cleared mammary tissue from pubertal (6–7 week old) *Csf1r-EGFP* mice. K8 immunostaining reveals the luminal cell layer; SMA marks the basal cell layer and SMA-positive vessels. **(A)** terminal end buds (TEBs), **(B)** ductal regions, **(C)** inguinal lymph node, and **(D)** nipple region. Arrows in **(A)** show Mϕs that have invaded the TEB epithelium and lumen (arrowhead). Arrow in **(B)** shows a Mϕ positioned between the epithelial bilayer. T, ductal tips; Du, ducts; LN, lymph node. Images are representative of 3 mice. **(E)** Second harmonic generation (SHG) showing fibrillar collagens around a TEB structure. Image stacks in middle panel are depth-coded (R-Y-G-C-B). Dashed arrow shows direction of TEB growth. Arrowhead in **(E)** shows a Mϕ interacting with collagen.

3D imaging of mammary tissue from pubertal *Csf1r-EGFP* mice revealed that mammary TEBs were enveloped by Mϕs, with spatial clustering observed (Figure 2A and Supplementary Figure S4A). Previous studies using the F4/80 marker indicated that Mϕs were mainly distributed at the neck of TEBs, whereas eosinophils (distinguished by their eosinic cytoplasm and bi-lobed nuclei) were concentrated at the TEB head (Gouon-Evans et al., 2000, 2002). By contrast, in this study GFP^+^ Mϕs in both locations shared stellate morphology (Figure 2A and Supplementary Figure S4A) and neither showed any evidence of segmented nuclei (Supplementary Figure S4A). A small number of mammary Mϕs were observed inside the body of TEBs (Figure 2A), where they may contribute to clearance of apoptotic cells from the TEB lumen (Humphreys et al., 1996; Gouon-Evans et al., 2000; Paine and Lewis, 2017). GFP^+^ Mϕs were found along the length of the ductal epithelium in the pubertal gland (Figure 2B and Supplementary Figure S4B) and in some cases appeared to be positioned between the luminal and basal cell layers (Figure 2B, arrow). Intraepithelial Mϕs, detected with F4/80, are a feature of ductal epithelia throughout the body (Hume D. A. et al., 1984). It is currently unclear how these interposed Mϕs affect luminal-basal cell connections [e.g., desmosomes and gap junctions (Shamir and Ewald, 2015)] and their precise function within the epithelial bilayer. GFP^+^ cells were also dispersed throughout the mammary fat pad (Figure 2 and Supplementary Figure S4; Schwertfeger et al., 2006; Chua et al., 2010) and were densely packed in the inguinal lymph node (Figure 2C and Supplementary Figure S4B) and nipple region (Figure 2D).

Mammary Mϕs have been shown to organize collagen into fibrillar bundles to steer TEB growth through the stromal fat pad (Ingman et al., 2006). We therefore examined fibrillar collagens with SHG (Williams et al., 2005) in tissue from *Csf1r-EGFP* mice at depth using an immersion-based optical clearing approach, which preserves endogenous fluorescence and tissue architecture (Lloyd-Lewis et al., 2016; Vigouroux et al., 2017). Although surface collagen fibers in the mammary gland were dense and multi-directional [Figure 2E (red)], deeper collagen fibers proximal to the growing TEB were aligned along its perimeter, extended in the direction of TEB growth and were associated with Mϕs (Figure 2E). These data provide further evidence that mechanical forces from the stroma guide epithelial development in the normal mammary gland (Ingman et al., 2006; Stewart et al., 2019).

### Mϕs Are Intimately Associated With the Mature Ductal Epithelium

**FIGURE 3:**
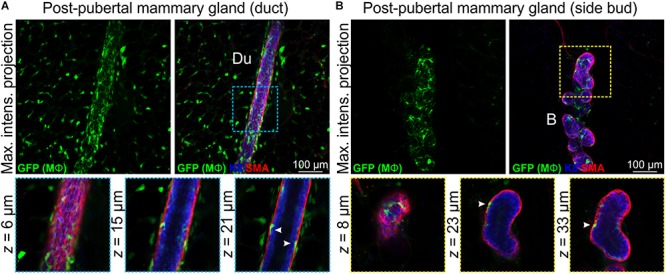
Mϕs in the mammary glands of post-pubertal virgin mice. Maximum intensity *z*-projection and single optical (*z*) slices of cleared mammary tissue from post-pubertal (12 week-old) *Csf1r-EGFP* mice. K8 immunostaining shows luminal cells; SMA immunostaining reveals basal cells and SMA-positive vessels. **(A)** Mammary ducts and **(B)** side buds. Du, duct; B, side bud. Arrowheads show Mϕs that are positioned within the epithelial bilayer. K8 immunostaining reveals the luminal cell layer and SMA marks the basal cell layer. Images are representative of 3 mice.

Mϕs are present in the post-pubertal mouse mammary gland at all phases of the estrus cycle, with the numbers being highest in diestrus (Chua et al., 2010). In tissue sections at all estrus stages, F4/80^+^ cells are detectable around alveolar side buds versus ducts, where they are thought to promote the development and regression of these transient structures (Chua et al., 2010). Using 3D imaging of mammary tissue from *Csf1r-EGFP* mice, we observed similar numbers of Mϕs closely associated with mammary ducts (Figure 3A and Supplementary Figure S5) and side buds (Figure 3B and Supplementary Figure S5A). As in the pubertal epithelium, Mϕs were also positioned between the luminal and basal cell layers in mature ducts and buds (Figures 3A,B and Supplementary Figure S5B, arrowheads) with some evidence of periodicity in intraepithelial Mϕ placement (Supplementary Figure S5B). This is consistent with regular distributions of Mϕs in many locations throughout the body (Hume D. et al., 2019). SHG of mature ducts revealed some fibrillar collagens that were located around the ducts and vessels (Supplementary Figure S5C).

### Mϕs Surround Alveolar Units in Gestation and Lactation

Mϕ deficient *Csf1^*op*^/Csf1^*op*^* female mice have compromised fertility (Pollard et al., 1991). Amongst those that do generate offspring, none are able to nurture a full litter, despite normal maternal behaviors (Pollard and Hennighausen, 1994). In-depth analyses of mammary tissue from pregnant and lactating *Csf1^*op*^/Csf1^*op*^* mice showed incomplete branching and precocious alveolar development (Pollard and Hennighausen, 1994) and F4/80^+^ cells have been detected around the developing and functional alveolar units during pregnancy and late gestation (Gouon-Evans et al., 2002).

**FIGURE 4:**
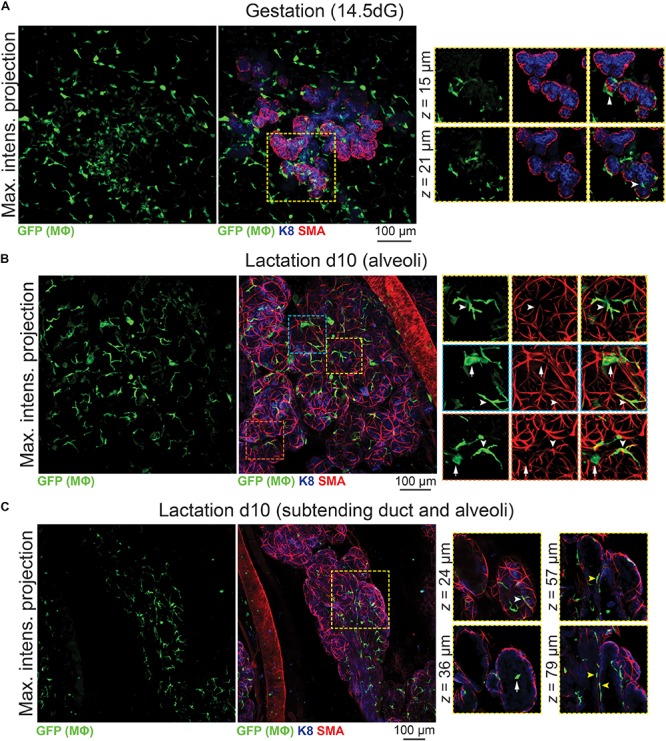
Mϕs in the mammary glands of pregnant and lactating mice. Maximum intensity *z*-projection and single optical (*z*) slices of cleared mammary tissue from **(A)** pregnant (14.5 days gestation, dG) and **(B,C)** lactating (day 10 lactation, d10) *Csf1r-EGFP* mice. K8 immunostaining reveals K8-positive luminal cells; smooth muscle actin (SMA) marks the basal/myoepithelial cells and SMA-positive vessels. Arrowheads in **(A)** show Mϕs that are interacting with the developing alveolar epithelium. In **(B,C)**, white arrowheads show Mϕs that are aligned along basal cells (versus white arrows showing Mϕs that are not imitating basal cell morphology). Yellow arrowheads in **(C)** show Mϕs that are positioned between the ductal epithelial bilayer. Images are representative of 3 mice at each developmental stage.

3D analysis of mammary tissue from pregnant *Csf1r-EGFP* mice (day 14.5 gestation, dG) confirmed Mϕ localization around the expanding alveolar structures (Figure 4A and Supplementary Figure S6). By lactation, Mϕs were observed immediately adjacent to alveolar basal cells, where they frequently imitated basal cell morphology (Figures 4B,C, white arrowheads). Mϕs were also present within lactational alveoli (Figure 4C, arrow), consistent with their enrichment in breast milk (Field, 2005).

### The Irreversible Phase of Involution Is Associated With an Increase in Mϕ Number in and Around Regressing Alveolar Structures

The number of Mϕs surrounding the mammary epithelium increases drastically from days 3–4 of involution (Lund et al., 1996; Stein et al., 2004; Hughes et al., 2012), and involution-associated Mϕs appear polarized toward tissue repair (O’Brien et al., 2010). The recruitment and polarization of Mϕs in the involuting mammary gland is regulated by epithelial *Stat3* expression (Hughes et al., 2012). Moreover, pre-weaning depletion of CSF1R-expressing cells reduces mammary epithelial cell death during post-lactational involution, an effect that can be reversed by orthotopic transplantation of bone marrow-derived Mϕs (O’Brien et al., 2012).

**FIGURE 5:**
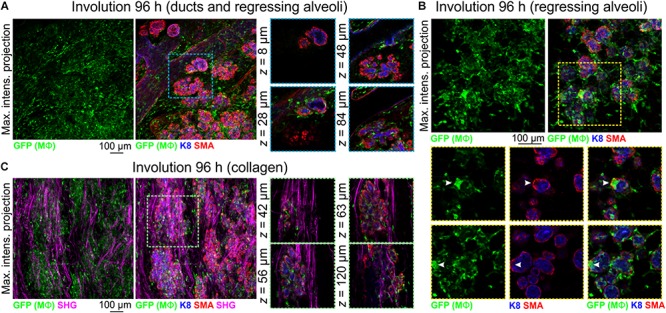
Mϕs in the mammary glands of mice during post-lactational involution. **(A–C)** Maximum intensity *z*-projection and single optical (*z*) slices of cleared mammary tissue from *Csf1r-EGFP* mice during involution (96 h post forced weaning). K8 immunostaining shows luminal cells; SMA immunostaining reveals basal cells and SMA-positive vessels. Arrowheads in **(B)** show a cluster of GFP^+^ Mϕs inside of collapsed alveolar units. **(C)** SHG showing fibrillar collagens surrounding regressing alveoli. Images are representative of 3 mice.

To further examine Mϕ number, morphology and distribution in the regressing mammary gland in 3-dimensions, we analyzed optically clear tissue from *Csf1r-EGFP* mice during the irreversible phase of involution. Relative to other developmental stages, Mϕ density was high at 96 h involution and Mϕs were observed around and inside ducts and regressing alveoli (Figures 5A,B). Large aggregates of GFP^+^ cells, reminiscent of homotypic fusion (MacLauchlan et al., 2009), were also observed inside degenerating alveolar structures (Figure 5B arrowheads). Similar aggregates of GFP^+^ Mϕs have been observed in a model of epithelial regeneration in the kidney following transient ischemia (Joo et al., 2016).

Collagen density increases during mammary gland involution and partially degraded non-fibrillar collagens have been suggested to be chemotactic for Mϕs (O’Brien et al., 2010). Intra- and interlobular fibrillar collagens were observed with SHG in *Csf1r-EGFP* mice and GFP^+^ Mϕs were observed to be associated with collagen fibrils (Figure 5C).

## Discussion

**FIGURE 6:**
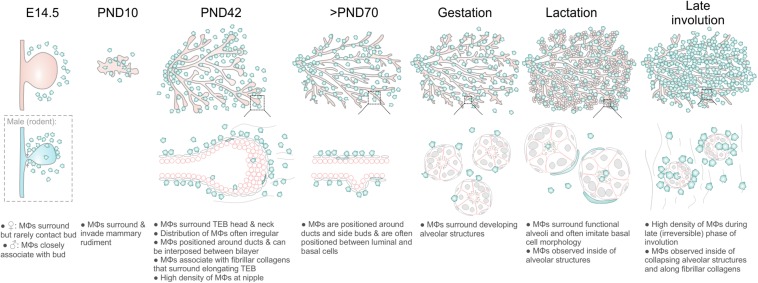
Diagram summarizing Mϕ distribution in the mouse mammary gland during distinct phases of development and remodeling.

Mϕs contribute to mammary gland development and remodeling at all developmental stages (Pollard and Hennighausen, 1994; Gouon-Evans et al., 2000; Dai et al., 2002; Van Nguyen and Pollard, 2002; Ingman et al., 2006; Chua et al., 2010; O’Brien et al., 2010, 2012; Hughes et al., 2012). The exact mechanisms by which tissue Mϕs regulate these processes are still being elucidated (Schwertfeger et al., 2006) and may be linked to their phagocytic, trophic and/or matrix remodeling functions (Sternlicht, 2006; Pollard, 2009). A comprehensive characterization of the stage-specific physiological roles of Mϕs in the mammary gland depends upon knowledge of their precise anatomical location within this organ. In this study, we provide new insights into the allocation, morphology and distribution of Mϕs in the embryonic, pre-pubertal, pubertal, post-pubertal, pregnant, lactating and involuting mammary glands of fluorescent reporter-positive mice *in situ* in 3-dimensions (Figure 6). Our study yields a number of important observations that could only be revealed by multi-dimensional imaging using a tamoxifen-independent, cell type-specific fluorescent reporter model (Hume D. et al., 2019; Hume D. A. et al., 2019). Firstly, in contrast to previous reports (Gouon-Evans et al., 2000, 2002), we demonstrate that Mϕs are not concentrated at the TEB neck, although some polarity in their distribution around TEBs was observed. These findings suggest that Mϕs may regulate mammary epithelial cells both within the head and neck of the TEB structure (Paine et al., 2016). Studies performing intravital imaging of TEB dynamics in *Csf1r-EGFP* mice are an aim for the future and may help to reveal possible correlations between Mϕ density and TEB behavior (e.g., turning and bifurcation events).

Mammary Mϕs were also frequently embedded between luminal and basal cells of the ductal epithelium. This has previously been observed in mammalian ductal epithelia, including the bile duct, salivary gland, tracheobronchial gland and mammary gland using thin sections prepared from formalin-fixed paraffin-embedded or frozen tissue (Hume D. A. et al., 1984; Sun et al., 2013). Regularity in the spacing of these intraepithelial Mϕs was also noted, which may arise through mutual repulsion (Hume D. et al., 2019) and could potentially contribute to regular distribution of adjacent populations of heterogeneous luminal and basal cells (Ismail et al., 2002; Davis et al., 2016). In sum, the work presented here suggests a close functional relationship between Mϕs and ductal epithelial cells, and possible communication between morphologically related Mϕ populations. Further studies are needed to determine whether these intraepithelial Mϕs share similar gene and protein expression patterns and whether this information can be used to probe their function, retention and passage within the epithelium. Tissue Mϕs have been shown to be influenced by properties of their specific niche within each tissue (e.g., anchoring scaffolds and local cues) (Chakarov et al., 2019; Mondor et al., 2019). Single cell sequencing of isolated mammary Mϕs from *Csf1r-EGFP* mice at distinct developmental stages, as exemplified by recent studies of other tissues (Chakarov et al., 2019; Mondor et al., 2019), might help to reveal the extent of functional diversity within Mϕ populations in this organ.

We reveal that Mϕs alter their morphology at distinct developmental stages, including the transition from gestation to lactation. The localization of Mϕs around growing alveolar units during gestation and the observation that Mϕ-deficient *Csf1^*op*^/Csf1^*op*^* mice exhibit precocious alveolar development, suggests that during this phase, alveolar-associated Mϕs may restrain alveologenesis. By analogy, Mϕs in the diaphragm appear to constrain the growth of lymphatic vessels and *Csf1r* mutation promoted branch formation of lymphatic sprouts (Ochsenbein et al., 2016). During lactation, Mϕs altered their anatomical position and were observed to closely imitate the morphology of adjacent, differentiated alveolar basal cells. Whether these cells specifically align themselves with oxytocin-responsive basal cells during lactation to modify basal cell function (Davis et al., 2015; Stevenson et al., 2019) or more simply to occupy the physical space that these force-exerting cells create within the alveolar epithelium (Davis, 2016; Stewart et al., 2019), remains to be seen. Such a function might be analogous to the role of a distinct population of CSF1-dependent Mϕs in the regulation of peristalsis in the muscularis externa of the intestine (Muller et al., 2014). Interestingly, in this study muscularis Mϕs and intestinal motility could be reversibly modified by lumen factors (Muller et al., 2014). Whether mammary Mϕs, positioned alongside alveolar basal cells, are capable of sampling the alveolar lumen environment to constrain basal cell-mediated alveolar contractility (e.g., in mastitis) has not yet been determined. Another possibility is that basal cell contractility may instead alter the function of alveolar Mϕs. Such an effect has been observed in the lung, another organ that is subject to cyclical mechanical stimulation, although this phenomenon was restricted to newly recruited monocytes and not the population of resident alveolar Mϕs (Solis et al., 2019). Finally, we were able to visualize for the first time tissue-resident Mϕs in the mesenchyme surrounding the mammary epithelial bud in 14.5 day-old female embryos. Intriguingly, these embryonic Mϕs rarely contacted the epithelial cells of the developing mammary bud at this stage of embryogenesis. This is in striking contrast to epithelial-Mϕ interactions in the early postnatal period, where Mϕs surround and invade the rudimentary ductal epithelium. This also contrasts with the male embryo, where Mϕs were often observed to both contact and infiltrate the epithelial bud at the time when its connection to the overlying epidermis is severed and the structure begins to regress (Dunbar et al., 1999; Heuberger et al., 2006; Cowin and Wysolmerski, 2010). At this stage, Mϕs may have an important role in clearing apoptotic epithelial and mesenchymal cells (Dunbar et al., 1999; Henson and Hume, 2006).

Mammary stem/progenitor cells are located within the mammary bud (in the embryo) and TEBs (in puberty). After ductal elongation is complete and TEBs regress, however, the location of long-lived mammary stem/progenitor cells and their putative niche remains unknown, although it has been suggested that these cells are deposited along the ductal epithelium by elongating TEBs (Davis et al., 2016; Lloyd-Lewis et al., 2017). In the 14.5 day embryo, Mϕs were positioned uniformly around, but not in contact with, the mammary bud. These data suggest that if a mammary stem cell-macrophage niche exists in the embryo around the time of lineage segregation, it operates over the scale of tens of micrometers and is fairly homogeneous. Mϕs were also positioned around pubertal TEBs, however, in contrast to the embryo, these cells contacted and infiltrated TEBs, were more densely arranged around these structures and often showed spatial clustering. Future studies combining tamoxifen-independent *Dll1-mCherry* (Chakrabarti et al., 2018) and *Csf1r-EGFP* mouse models with optical tissue clearing and 3D imaging may help to reveal the precise location of mammary stem/progenitor cells within TEBs and the post-pubertal ductal epithelium. It should be noted, however, that whilst ductal elongation is delayed in *Csf1^*op*^/Csf1^*op*^* mice, these structures are still capable of invading the fat pad and by 12 weeks of age have reached the fat pad limits (Gouon-Evans et al., 2000). These findings imply that mammary epithelial cells have mechanisms to overcome insufficiencies in niche signaling. One candidate is the alternative CSF1R ligand, IL34, which may also be expressed by mammary epithelial cells (DeNardo et al., 2011). Studies investigating the activation and roles of the CSF1R in mammary development have been thwarted by the severe postnatal phenotype of *Csf1r^–^/Csf1r^–^* mice (Chitu and Stanley, 2017), but may be more amenable to study in recently described *Csf1r^–^/Csf1r^–^* rats (Pridans et al., 2019). Alternatively, these findings may reflect a long-term plasticity in mammary epithelial cells (Lilja et al., 2018) and a shifting definition of “stemness” in some tissues away from a unidirectional, top-down model to a model where stemness is considered as a cell state that may be acquired or extinguished under specific microenvironmental conditions (Laplane and Solary, 2019). A closer examination of mammary cell behaviors—including lineage segregation—under conditions of Mϕ depletion may provide important insights into epithelial plasticity in this vital mammalian organ.

## Data Availability Statement

All datasets generated for this study are included in the manuscript/Supplementary Files.

## Ethics Statement

The animal study was reviewed and approved by the University of Queensland Health Sciences Animal Ethics Committee. In accordance with the Australian Code for the Care and Use of Animals for Scientific Purposes and the Queensland Animal Care and Protection Act (2001).

## Author Contributions

FD and TS performed all the experiments. FD, DH, and TS, conceived and designed the experiments. TS, KH, DH, and FD analyzed the results. FD wrote the manuscript. DH, KH, and TS edited the manuscript.

## Conflict of Interest

The authors declare that the research was conducted in the absence of any commercial or financial relationships that could be construed as a potential conflict of interest.

## References

[B1] Boulanger J. Kervrann C. Bouthemy P. Elbau P. Sibarita J.-B. Salamero J. (2010). Patch-based nonlocal functional for denoising fluorescence microscopy image sequences. *IEEE Trans. Med. Imaging* 29 442–454. 10.1109/TMI.2009.2033991 19900849

[B2] Castellana D. Paus R. Perez-Moreno M. (2014). Macrophages contribute to the cyclic activation of adult hair follicle stem cells. *PLoS Biol.* 12:e1002002. 10.1371/journal.pbio.1002002 25536657 PMC4275176

[B3] Chakarov S. Lim H. Y. Tan L. Lim S. Y. See P. Lum J. (2019). Two distinct interstitial macrophage populations coexist across tissues in specific subtissular niches. *Science* 363:eaau0964. 10.1126/science.aau0964 30872492

[B4] Chakrabarti R. Celià-Terrassa T. Kumar S. Hang X. Wei Y. Choudhury A. (2018). Notch ligand Dll1 mediates cross-talk between mammary stem cells and the macrophageal niche. *Science* 360:eaan4153. 10.1126/science.aan4153 29773667 PMC7881440

[B5] Chitu V. Stanley E. R. (2017). Regulation of embryonic and postnatal development by the CSF-1 receptor. *Curr. Top. Dev. Biol.* 123 229–275. 10.1016/bs.ctdb.2016.10.004 28236968 PMC5479137

[B6] Chua A. C. L. Hodson L. J. Moldenhauer L. M. Robertson S. A. Ingman W. V. (2010). Dual roles for macrophages in ovarian cycle-associated development and remodelling of the mammary gland epithelium. *Development* 137 4229–4239. 10.1242/dev.059261 21068060

[B7] Cowin P. Wysolmerski J. (2010). Molecular mechanisms guiding embryonic mammary gland development. *Cold Spring Harb. Perspect. Biol.* 2:a003251. 10.1101/cshperspect.a003251 20484386 PMC2869520

[B8] Dai X. M. Ryan G. R. Hapel A. J. Dominguez M. G. Russell R. G. Kapp S. (2002). Targeted disruption of the mouse colony-stimulating factor 1 receptor gene results in osteopetrosis, mononuclear phagocyte deficiency, increased primitive progenitor cell frequencies, and reproductive defects. *Blood* 99 111–120. 10.1182/blood.V99.1.111 11756160

[B9] Davis F. M. (2016). The ins and outs of calcium signalling in lactation and involution: implications for breast cancer treatment. *Pharmacol. Res.* 116 100–104. 10.1016/j.phrs.2016.12.007 27965034

[B10] Davis F. M. Janoshazi A. Janardhan K. S. Steinckwich N. D’Agostin D. M. Petranka J. G. (2015). Essential role of orai1 store-operated calcium channels in lactation. *Proc. Natl. Acad. Sci. U.S.A.* 112 5827–5832. 10.1073/pnas.1502264112 25902527 PMC4426473

[B11] Davis F. M. Lloyd-Lewis B. Harris O. B. Kozar S. Winton D. J. Muresan L. (2016). Single-cell lineage tracing in the mammary gland reveals stochastic clonal dispersion of stem/progenitor cell progeny. *Nat. Commun.* 7:13053. 10.1038/ncomms13053 27779190 PMC5093309

[B12] DeNardo D. G. Brennan D. J. Rexhepaj E. Ruffell B. Shiao S. L. Madden S. F. (2011). Leukocyte complexity predicts breast cancer survival and functionally regulates response to chemotherapy. *Cancer Discov.* 1 54–67. 10.1158/2159-8274.CD-10-0028 22039576 PMC3203524

[B13] Dunbar M. E. Dann P. R. Zhang J.-P. Wysolmerski J. J. Robinson G. W. Hennighausen L. (1999). Parathyroid hormone-related protein signaling is necessary for sexual dimorphism during embryonic mammary development. *Development* 126 3485–3493. 10409496 10.1242/dev.126.16.3485

[B14] Field C. J. (2005). The immunological components of human milk and their effect on immune development in infants. *J. Nutr.* 135 1–4. 10.1093/jn/135.1.1 15623823

[B15] Gouon-Evans V. Lin E. Y. Pollard J. W. (2002). Requirement of macrophages and eosinophils and their cytokines/chemokines for mammary gland development. *Breast Cancer Res.* 4 155–164. 10.1186/bcr441 12100741 PMC138736

[B16] Gouon-Evans V. Rothenberg M. E. Pollard J. W. (2000). Postnatal mammary gland development requires macrophages and eosinophils. *Development* 127 2269–2282. 10804170 10.1242/dev.127.11.2269

[B17] Gyorki D. E. Asselin-Labat M. L. van Rooijen N. Lindeman G. J. Visvader J. E. (2009). Resident macrophages influence stem cell activity in the mammary gland. *Breast Cancer Res.* 11:R62. 10.1186/bcr2353 19706193 PMC2750124

[B18] Henson P. M. Hume D. A. (2006). Apoptotic cell removal in development and tissue homeostasis. *Trends Immunol.* 27 244–250. 10.1016/j.it.2006.03.005 16584921

[B19] Heuberger B. Fitzka I. Wasner G. Kratochwil K. (2006). Induction of androgen receptor formation by epithelium-mesenchyme interaction in embryonic mouse mammary gland. *Proc. Natl. Acad. Sci. U.S.A.* 79 2957–2961. 10.1073/pnas.79.9.2957 6953441 PMC346327

[B20] Hodson L. J. Chua A. C. L. Evdokiou A. Robertson S. A. Ingman W. V. (2013). Macrophage phenotype in the mammary gland fluctuates over the course of the estrous cycle and is regulated by ovarian steroid hormones1. *Biol. Reprod.* 89 1–8. 10.1095/biolreprod.113.109561 23926283

[B21] Hughes K. Wickenden J. A. Allen J. E. Watson C. J. (2012). Conditional deletion of Stat3 in mammary epithelium impairs the acute phase response and modulates immune cell numbers during post-lactational regression. *J. Pathol.* 227 106–117. 10.1002/path.3961 22081431 PMC3477635

[B22] Hume D. A. (2015). The many alternative faces of macrophage activation. *Front. Immunol.* 6:370. 10.3389/fimmu.2015.00370 26257737 PMC4510422

[B23] Hume D. A. Caruso M. Ferrari-Cestari M. Summers K. M. Pridans C. Irvine K. M. (2019). Phenotypic impacts of CSF1R deficiencies in humans and model organisms. *J. Leukoc. Biol.* 10.1002/JLB.MR0519-143R [Epub ahead of print]. 31330095

[B24] Hume D. Irvine K. Pridans C. (2019). The mononuclear phagocyte system: the relationship between monocytes and macrophages. *Trends Immunol.* 40 98–112. 10.1016/j.it.2018.11.007 30579704

[B25] Hume D. A. Perry V. H. Gordon S. (1984). The mononuclear phagocyte system of the mouse defined by immunohistochemical localisation of antigen F4/80: macrophages associated with epithelia. *Anat. Rec.* 210 503–512. 10.1002/ar.1092100311 6524692

[B26] Humphreys R. C. Krajewska M. Krnacik S. Jaeger R. Weiher H. Krajewski S. (1996). Apoptosis in the terminal endbud of the murine mammary gland: a mechanism of ductal morphogenesis. *Development* 122 4013–4022. 9012521 10.1242/dev.122.12.4013

[B27] Ingman W. V. Wyckoff J. Gouon-Evans V. Condeelis J. Pollard J. W. (2006). Macrophages promote collagen fibrillogenesis around terminal end buds of the developing mammary gland. *Dev. Dyn.* 235 3222–3229. 10.1002/dvdy.20972 17029292

[B28] Ismail P. M. Li J. DeMayo F. J. O’Malley B. W. Lydon J. P. (2002). A novel LacZ reporter mouse reveals complex regulation of the progesterone receptor promoter during mammary gland development. *Mol. Endocrinol.* 16 2475–2489. 10.1210/me.2002-2169 12403837

[B29] Jäppinen N. Félix I. Lokka E. Tyystjärvi S. Pynttäri A. Lahtela T. (2019). Fetal-derived macrophages dominate in adult mammary glands. *Nat. Commun.* 10:281. 10.1038/s41467-018-08065-8061 30655530 PMC6336770

[B30] Joo S. Kim D. K. Sim H. J. Lee G. D. Hwang S. K. Choi S. (2016). Clinical results of sublobar resection versus lobectomy or more extensive resection for lung cancer patients with idiopathic pulmonary fibrosis. *J. Thorac. Dis.* 8 977–984. 10.21037/jtd.2016.03.76 27162674 PMC4842788

[B31] Kannan N. Eaves C. J. (2018). Macrophages stimulate mammary stem cells. *Science* 360 1401–1402. 10.1126/science.aau1394 29954968 PMC6162096

[B32] Laplane L. Solary E. (2019). Philosophy of biology: towards a classification of stem cells. *eLife* 8:e46563. 10.7554/eLife.46563 30864951 PMC6415933

[B33] Lilja A. M. Rodilla V. Huyghe M. Hannezo E. Landragin C. Renaud O. (2018). Clonal analysis of Notch1-expressing cells reveals the existence of unipotent stem cells that retain long-term plasticity in the embryonic mammary gland. *Nat. Cell Biol.* 20 677–687. 10.1038/s41556-018-0108-1 29784917 PMC6984964

[B34] Linkert M. Rueden C. T. Allan C. Burel J. M. Moore W. Patterson A. (2010). Metadata matters: access to image data in the real world. *J. Cell Biol.* 189 777–782. 10.1083/jcb.201004104 20513764 PMC2878938

[B35] Lloyd-Lewis B. Davis F. M. Harris O. B. Hitchcock J. R. Lourenco F. C. Pasche M. (2016). Imaging the mammary gland and mammary tumours in 3D: optical tissue clearing and immunofluorescence methods. *Breast Cancer Res.* 18:127. 27964754 10.1186/s13058-016-0754-9 PMC5155399

[B36] Lloyd-Lewis B. Davis F. M. Harris O. B. Hitchcock J. R. Watson C. J. (2018). Neutral lineage tracing of proliferative embryonic and adult mammary stem/progenitor cells. *Development* 145:dev164079. 10.1242/dev.164079 30045917 PMC6078330

[B37] Lloyd-Lewis B. Harris O. B. Watson C. J. Davis F. M. (2017). Mammary stem cells: premise, properties and perspectives. *Trends Cell Biol.* 8 556–567. 10.1016/j.tcb.2017.04.001 28487183

[B38] Lund L. R. Rømer J. Thomasset N. Solberg H. Pyke C. Bissell M. J. (1996). Two distinct phases of apoptosis in mammary gland involution: proteinase-independent and -dependent pathways. *Development* 122 181–193. 10.1111/j.1600-6143.2008.02497.x.Plasma 8565829 PMC2933211

[B39] MacLauchlan S. Skokos E. A. Meznarich N. Zhu D. H. Raoof S. Shipley J. M. (2009). Macrophage fusion, giant cell formation, and the foreign body response require matrix metalloproteinase 9. *J. Leukoc. Biol.* 85 617–626. 10.1189/jlb.1008588 19141565 PMC2656428

[B40] Mondor I. Baratin M. Lagueyrie M. Saro L. Henri S. Gentek R. (2019). Lymphatic endothelial cells are essential components of the subcapsular sinus macrophage niche. *Immunity* 50 1453–1466. 10.1016/j.immuni.2019.04.002 31053503 PMC6697131

[B41] Muller P. A. Koscsó B. Rajani G. M. Stevanovic K. Berres M. L. Hashimoto D. (2014). Crosstalk between muscularis macrophages and enteric neurons regulates gastrointestinal motility. *Cell* 158 300–313. 10.1016/j.cell.2014.04.050 25036630 PMC4149228

[B42] Naik S. Larsen S. B. Cowley C. J. Fuchs E. (2018). Two to tango: dialog between immunity and stem cells in health and disease. *Cell* 175 908–920. 10.1016/j.cell.2018.08.071 30388451 PMC6294328

[B43] O’Brien J. Lyons T. Monks J. Lucia M. S. Wilson R. S. Hines L. (2010). Alternatively activated macrophages and collagen remodeling characterize the postpartum involuting mammary gland across species. *Am. J. Pathol.* 176 1241–1255. 10.2353/ajpath.2010.090735 20110414 PMC2832146

[B44] O’Brien J. Martinson H. Durand-Rougely C. Schedin P. (2012). Macrophages are crucial for epithelial cell death and adipocyte repopulation during mammary gland involution. *Development* 139 269–275. 10.1242/dev.071696 22129827 PMC13621425

[B45] Ochsenbein A. M. Karaman S. Proulx S. T. Goldmann R. Chittazhathu J. Dasargyri A. (2016). Regulation of lymphangiogenesis in the diaphragm by macrophages and VEGFR-3 signaling. *Angiogenesis* 19 513–524. 10.1007/s10456-016-9523-9528 27464987 PMC5026726

[B46] Paine I. Chauviere A. Landua J. Sreekumar A. Cristini V. Rosen J. (2016). A geometrically-constrained mathematical model of mammary gland ductal elongation reveals novel cellular dynamics within the terminal end bud. *PLoS Comput. Biol.* 12:e1004839. 10.1371/journal.pcbi.1004839 27115287 PMC4845990

[B47] Paine I. S. Lewis M. T. (2017). The terminal end bud: the little engine that could. *J. Mammary Gland Biol. Neoplasia* 22 93–108. 10.1007/s10911-017-9372-9370 28168376 PMC5488158

[B48] Pollard J. W. (2009). Trophic macrophages in development and disease. *Nat. Rev. Immunol.* 9 259–270. 10.1038/nri2528 19282852 PMC3648866

[B49] Pollard J. W. Hennighausen L. (1994). Colony stimulating factor 1 is required for mammary gland development during pregnancy. *Proc. Natl. Acad. Sci. U.S.A.* 91 9312–9316. 10.1073/pnas.91.20.9312 7937762 PMC44802

[B50] Pollard J. W. Hunt J. S. Wiktor-Jedrzejczak W. Stanley E. R. (1991). A pregnancy defect in the osteopetrotic (op op) mouse demonstrates the requirement for CSF-1 in female fertility. *Dev. Biol.* 148 273–283. 10.1016/0012-1606(91)90336-90332 1834496

[B51] Pridans C. Raper A. Davis G. M. Alves J. Sauter K. A. Lefevre L. (2019). Pleiotropic impacts of macrophage and microglial deficiency on development in rats with targeted mutation of the Csf1r locus. *J. Immunol.* 201 2683–2699. 10.4049/jimmunol.1900420 PMC6196293 30249809

[B52] Sargeant T. J. Lloyd-Lewis B. Resemann H. K. Ramos-Montoya A. Skepper J. Watson C. J. (2014). Stat3 controls cell death during mammary gland involution by regulating uptake of milk fat globules and lysosomal membrane permeabilization. *Nat. Cell Biol.* 16 1057–1068. 10.1038/ncb3043 25283994 PMC4216597

[B53] Sasmono R. T. Oceandy D. Pollard J. W. Tong W. Pavli P. Wainwright B. J. (2003). A macrophage colony-stimulating factor receptor-green fluorescent protein transgene is expressed throughout the mononuclear phagocyte system of the mouse. *Blood* 101 1155–1163. 10.1182/blood-2002-02-0569 12393599

[B54] Schindelin J. Arganda-Carreras I. Frise E. Kaynig V. Longair M. Pietzsch T. (2012). Fiji: an open source platform for biological image analysis. *Nat. Methods* 9 676–682. 10.1038/nmeth.2019 22743772 PMC3855844

[B55] Schwertfeger K. L. Rosen J. M. Cohen D. A. (2006). Mammary gland macrophages: pleiotropic functions in mammary development. *J. Mammary Gland Biol. Neoplasia* 11 229–238. 10.1007/s10911-006-9028-y 17115264

[B56] Sehgal A. Donaldson D. S. Pridans C. Sauter K. A. Hume D. A. Mabbott N. A. (2018). The role of CSF1R-dependent macrophages in control of the intestinal stem-cell niche. *Nat. Commun.* 9:1272. 10.1038/s41467-018-03638-3636 29593242 PMC5871851

[B57] Shamir E. R. Ewald A. J. (2015). Adhesion in mammary development: novel roles for E-cadherin in individual and collective cell migration. *Curr. Top. Dev. Biol.* 112 353–382. 10.1016/bs.ctdb.2014.12.001 25733146 PMC4696070

[B58] Solis A. G. Bielecki P. Steach H. R. Sharma L. Harman C. C. D. Yun S. (2019). Mechanosensation of cyclical force by PIEZO1 is essential for innate immunity. *Nature* 573 69–74. 10.1038/s41586-019-1485-1488 31435009 PMC6939392

[B59] Sreekumar A. Roarty K. Rosen J. M. (2015). The mammary stem cell hierarchy: a looking glass into heterogeneous breast cancer landscapes. *Endocr. Relat. Cancer* 22 T161–T176. 10.1530/ERC-15-0263 26206777 PMC4618079

[B60] Stein T. Morris J. S. Davies C. R. Weber-Hall S. J. Duffy M.-A. Heath V. J. (2004). Involution of the mouse mammary gland is associated with an immune cascade and an acute-phase response, involving LBP, CD14 and STAT3. *Breast Cancer Res.* 6:R75. 10.1186/bcr753 14979920 PMC400652

[B61] Sternlicht M. D. (2006). Key stages in mammary gland development: the cues that regulate ductal branching morphogenesis. *Breast Cancer Res.* 8:201. 10.1186/bcr1368 16524451 PMC1413974

[B62] Stevenson A. J. Vanwalleghem G. Stewart T. A. (2019). *Multiscale Activity Imaging in the Mammary Gland Reveals How Oxytocin Enables Lactation. Biorxiv*. [Preprint] Available at: https://www.terkko.helsinki.fi/article/20877300_multiscale-activity-imaging-in-the-mammary-gland-reveals-how-oxytocin-enables-lactation (accessed August 21, 2019).

[B63] Stewart T. A. Hughes K. Stevenson A. S. J. Marino N. Ju A. J. L. Morehead M. (2019). *Mammary Mechanobiology: Mechanically-Activated Ion Channels in Lactation and Involution. BioRxiv.* [Preprint] 10.1101/649038 33262312

[B64] Sun X. Robertson S. A. Ingman W. V. (2013). Regulation of epithelial cell turnover and macrophage phenotype by epithelial cell-derived transforming growth factor beta1 in the mammary gland. *Cytokine* 61 377–388. 10.1016/j.cyto.2012.12.002 23290315

[B65] Susaki E. A. Tainaka K. Perrin D. Kishino F. Tawara T. Watanabe T. M. (2014). Whole-brain imaging with single-cell resolution using chemical cocktails and computational analysis. *Cell* 157 726–739. 10.1016/j.cell.2014.03.042 24746791

[B66] Van Nguyen A. Pollard J. W. (2002). Colony stimulating factor-1 is required to recruit macrophages into the mammary gland to facilitate mammary ductal outgrowth. *Dev. Biol.* 247 11–25. 10.1006/dbio.2002.0669 12074549

[B67] Vigouroux R. J. Belle M. Chédotal A. (2017). Neuroscience in the third dimension: shedding new light on the brain with tissue clearing. *Mol. Brain* 10:33. 10.1186/s13041-017-0314-y 28728585 PMC5520295

[B68] Watson C. J. Khaled W. T. (2008). Mammary development in the embryo and adult: a journey of morphogenesis and commitment. *Development* 135 995–1003. 10.1242/dev.005439 18296651

[B69] Williams R. M. Zipfel W. R. Webb W. W. (2005). Interpreting second-harmonic generation images of collagen I fibrils. *Biophys. J.* 88 1377–1386. 10.1529/biophysj.104.047308 15533922 PMC1305140

[B70] Winkler I. G. Sims N. A. Pettit A. R. Barbier V. Nowlan B. Helwani F. (2010). Bone marrow macrophages maintain hematopoietic stem cell (HSC) niches and their depletion mobilizes HSCs. *Blood* 116 4815–4828. 10.1182/blood-2009-11-253534 20713966

[B71] Wuidart A. Sifrim A. Fioramonti M. Matsumura S. Brisebarre A. Brown D. (2018). Early lineage segregation of multipotent embryonic mammary gland progenitors. *Nat. Cell Biol.* 20 666–676. 10.1038/s41556-018-0095-2 29784918 PMC5985933

[B72] Yang M. McKay D. Pollard J. W. Lewis C. E. (2018). Diverse functions of macrophages in different tumor microenvironments. *Cancer Res.* 78 5492–5503. 10.1158/0008-5472.CAN-18-1367 30206177 PMC6171744

